# Deciphering Obesity-Related Gene Clusters Unearths SOCS3 Immune Infiltrates and 5mC/m6A Modifiers in Ossification of Ligamentum Flavum Pathogenesis

**DOI:** 10.3389/fendo.2022.861567

**Published:** 2022-05-30

**Authors:** Baoliang Zhang, Lei Yuan, Guanghui Chen, Xi Chen, Xiaoxi Yang, Tianqi Fan, Chuiguo Sun, Dongwei Fan, Zhongqiang Chen

**Affiliations:** ^1^ Department of Orthopaedics, Peking University Third Hospital, Beijing, China; ^2^ Beijing Key Laboratory of Spinal Disease Research, Beijing, China; ^3^ Engineering Research Center of Bone and Joint Precision Medicine, Ministry of Education, Beijing, China

**Keywords:** ossification of ligamentum flavum, immune infiltration, obesity, inflammation, M6A, DNA methylation

## Abstract

**Background:**

Ossification of ligamentum flavum (OLF) is an insidious and debilitating heterotopic ossifying disease with etiological heterogeneity and undefined pathogenesis. Obese individuals predispose to OLF, whereas the underlying connections between obesity phenotype and OLF pathomechanism are not fully understood. Therefore, this study aims to explore distinct obesity-related genes and their functional signatures in OLF.

**Methods:**

The transcriptome sequencing data related to OLF were downloaded from the GSE106253 in the Gene Expression Omnibus (GEO) database. The obesity-related differentially expressed genes (ORDEGs) in OLF were screened, and functional and pathway enrichment analysis were applied for these genes. Furthermore, protein-protein interactions (PPI), module analysis, transcription factor enrichment analysis (TFEA), and experiment validation were used to identify hub ORDEGs. The immune infiltration landscape in OLF was depicted, and correlation analysis between core gene SOCS3 and OLF-related infiltrating immune cells (OIICs) as well as 5mC/m6A modifiers in OLF was constructed.

**Results:**

Ninety-nine ORDEGs were preliminarily identified, and functional annotations showed these genes were mainly involved in metabolism, inflammation, and immune-related biological functions and pathways. Integrative bioinformatic algorithms determined a crucial gene cluster associated with inflammatory/immune responses, such as TNF signaling pathway, JAK-STAT signaling pathway, and regulation of interferon-gamma-mediated signaling. Eight hub ORDEGs were validated, including 6 down-regulated genes (SOCS3, PPARG, ICAM-1, CCL2, MYC, and NT5E) and 2 up-regulated genes (PTGS2 and VEGFA). Furthermore, 14 differential OIICs were identified by ssGSEA and xCell, and SOCS3 was overlapped to be the core gene, which was associated with multiple immune infiltrates (dendritic cells, macrophage, and T cells) and six m6A modifiers as well as four 5mC regulators in OLF. Reduced SOCS3 and FTO expression and up-regulated DNMT1 level in OLF were validated by Western blotting.

**Conclusion:**

This study deciphered immune/inflammatory signatures of obesity-related gene clusters for the first time, and defined SOCS3 as one core gene. The crosstalk between 5mC/m6A methylation may be a key mediator of SOCS3 expression and immune infiltration. These findings will provide more insights into molecular mechanisms and therapeutic targets of obesity-related OLF.

## Introduction

Ossification of ligamentum flavum (OLF) is the major pathogeny of severe thoracic myelopathy characterized by abnormally heterotopic bone formation of the intraspinal ligament with unelucidated pathogenesis (1). To date, genetic, mechanical stress, degeneration, inflammatory, and metabolic factors have been found to be involved in the occurrence and progression of OLF (2–6). Epidemiological studies indicated that a clinical subset of patients with obesity were more susceptible to early-onset or even diffuse OLF (7). Additionally, concrete evidence has identified obesity as an independent causal factor for the development of OLF in non-elderly adults (8). A recent cross-sectional study also demonstrated the aggravation of OLF associated with the degree of obesity (9). These findings revealed obesity might not only act as a starter but also a facilitator of OLF. Moreover, our previous research has proven that leptin, an adipocyte-derived product encoded by the Ob gene, could induce significant osteogenic differentiation in ossified ligamentum flavum cells (OLFCs), but not in normal ligamentum flavum cells (NLFCs) (10). It is speculated that altered expression of specific genes might influence the responsiveness of ligamentum flavum cells to the obesity-induced persistent stimulus, such leptin. However, the underlying biochemical links between obesity and OLF pathogenesis have not been investigated. From this point of view, sophisticated bioinformatics algorithms for transcriptomics data were adopted for screening obesity-related deferentially expressed genes (ORDEGs) between normal and OLF samples, and analyzing their functional signatures, which may help lift the veil on the molecular mechanisms of obesity-related OLF.

Recently, epigenetics has been shown to have impact on human health and disease susceptibility (11, 12). On one hand, obesity can affect epigenetic marks such as DNA methylation (5mC), non-coding RNA, and RNA N6-methyladenosine methylation (m6A), thereby promoting changes in gene expression of critical genes associated with the pathophysiology of several obesity-related diseases (13–15). On the other hand, accumulating evidence indicated that epigenetic mechanism might be an important contributor to the pathogenesis of OLF (16–18). DNA methylation, one of the most common epigenetic modifications, can serve as a bridge between environmental changes and cellular responses, and our whole-genome DNA methylation sequencing analysis revealed distinct DNA methylation patterns in different types of OLF (17). In addition, m6A modification is the most prevalent and abundant internal modification on eukaryotic mRNAs, and a previous study has demonstrated BMP2 modified by the m6A demethylation enzyme ALKBH5 in OLF pathology (18). Notably, FTO is the first m6A demethylase that reduces m6A methylation and contributes to inflammation in obesity (19). Therefore, the identification of these epigenetic changes of specific genes is a recent and innovative field of research in OLF pathogenesis.

On this project, the mRNA microarray dataset GSE106253 from the Gene Expression Omnibus (GEO) was reinterpreted to screen potential ORDEGs by the R software, online website, and other databases. Then, the protein-protein interaction (PPI) and module analysis were constructed to further determine hub ORDEGs and annotate their potential biological functions. Next, transcription factor (TF) enrichment analysis, correlation analysis, co-expression analysis, and quantitative real-time polymerase chain reaction (qRT-PCR) were applied for the expression of key genes. Eventually, the correlation between the expression of SOCS3 and immune cell infiltration as well as 5mC/m6A regulators in OLF was explored, and promising 5mC/m6A modifiers related to SOCS3 expression were verified by Western blotting. These findings will provide a theoretical basis for discovering molecular mechanisms underlying obesity-related OLF.

## Methods

### Collection of OLF-Related Microarray Data and Human Obesity-Related Genes Dataset

According to the inclusion criteria (1): organism: Homo sapiens (2); expression profiling by microarray; and (3) samples: OLF ligament tissues and normal ligament tissues, an eligible high-throughput RNA-sequencing data (GSE106253) was downloaded from GEO for further analysis. The microarray data contains the mRNA information of ligamentum flavum tissue from 4 OLF patients and 4 healthy individuals. Meanwhile, all obesity-related genes (ORGs) in Homo sapiens were obtained from Integratomics TIME, GWAS, T-HOD, and KEGG PATHWAY databases.

### Identification of Obesity-Related Differentially Expressed Genes in OLF

The raw data from GSE106253 was reviewed for background correction and data normalization through the affy package of R software. Based on the predetermined statistical threshold of |fold change| > 1 and adjusted *P* < 0.05, differentially expressed genes (DEGs) between OLF samples and healthy controls were screened out through GEO2R, an interactive online tool. Furthermore, these DEGs and ORGs were intersected to obtain ORDEGs in OLF. Clustering heatmap and circular heatmap were performed to describe the expression of DEGs and ORDEGs, respectively.

### Functional and Pathway Enrichment Analysis of Up-Regulated and Down-Regulated ORDEGs in OLF

To comprehensively evaluate GO and biological pathways of ORDEGs, we applied different algorithms to perform mutual verification through different enrichment analysis tools, including g: Profiler, DAVID 6.8, Funrich, and WebGestalt. GO analysis was performed to illustrate their functions in the biology process (BP), cell component (CC), and molecular function (MF). Enrichment pathway analysis was conducted in three well renowned databases: Kyoto Encyclopedia of Genes and Genomes (KEGG), Reactome (REAC), and WikiPathways (WP). Gene set enrichment analysis (GSEA) is a method to sort and compare genes with predefined gene sets according to the degree of differential expression of two samples, so ORDEGs were uploaded to the GSEA for more accurate analysis.

### Construction of Protein-Protein Interaction (PPI) Network and Module Genetic Analysis

Search Tool for the Retrieval of Interacting Genes (STRING; http://string-db.org; version 11.5), an online database for predicting protein interactions, was applied to construct the PPI network of ORDEGs when interactions score > 0.4 were taken as statistically significant. Cytoscape (version 3.8.1) was used to visualize molecular interaction networks and analyze the capabilities of the PPI network under default parameters. The Network Analyzer plug-in was utilized to calculate important nodes of protein interactions within the network. A plug-in for Cytoscape, MCODE, was used to determine the hub gene and extract sub-networks based on the connectivity degree of genes to surrounding genes in the network.

### Functional Annotations of Key Genes From Identified Gene Clusters

Metascape was used to further verify the function enrichment of hub ORDEGs with *P* < 0.05 as the cutoff. Pathway analysis of hub genes was performed and visualized by ClueGO (version 2.5.8) and CluePedia (version 1.5.8). *P* < 0.01 was statistically significant. In addition, considering the obesity status is closely related to inflammation and immune response, the immune-related function annotation of hub ORDEGs was analyzed separately using ImmuneSystemProcess in ClueGO (version 2.5.8).

### Transcription Factors (TF) Enrichment Analysis, Gene Co-Expression Analysis and Correlation Analysis

TF enrichment analysis prioritizes transcription factors based on the overlap between given lists of differentially expressed genes, and previously annotated TF targets assembled from multiple resources. ChEA3 (https://maayanlab.cloud/chea3/) is a web-based TFEA tool to aid in identifying the TFs responsible for observed changes in gene expression with specific functions. GENEMANIA (http://genemania.org/search/) was used to construct a gene-gene interaction network for hub ORDEGs to evaluate the functions of these genes. The correlation analysis between gene-gene and series-series was also conducted.

### Correlation Analysis Between SOCS3 and OLF-Related Immune Infiltrating Cells (OIICs)

ssGSEA was introduced to calculate the relative infiltration scores of 23 different immune cell types based on the expression of reference genes within the gene sets from RNA-seq data. xCell, a novel gene signature-based method reliably portraying the cellular heterogeneity landscape of tissue expression profiles, was utilized to infer the estimated proportion of 64 types of immune cells. The cut-off values for the cell analyses were *P* < 0.05. The correlation between SOCS3 level and OIICs was evaluated. In addition, we analyzed the correlation between SOCS3 and immune cell markers to evaluate the potential role of SOCS3 in immunity. Immune cell markers were selected from the website of R&D Systems (www.rndsystems.com/cn/resources/cell-markers/immune-cells), including markers of B cells, T cells, CD8^+^ T cells, follicular helper T cells (Tfh), T-helper 1 (Th1) cells, T-helper 2 (Th2) cells, T-helper 17 (Th17) cells, Treg, T cells exhausted, M1 macrophages, M2 macrophages, monocytes, natural killer (NK) cells, neutrophils, and dendritic cells (DC).

### Correlation Analysis Between SOCS3 Expression and m6A Associated Genes in OLF

The R software package was utilized to evaluate the correlation between the expression of SOCS3 and the expression of 20 m6A modifiers in OLF, including “readers” ELAVL1, FMR1, YTHDC1, YTHDC2, IGF2BP1, IGF2BP2, YTHDF1, YTHDF2, YTHDF3, HNRNPA2B1, TRA2A, RBMX, “writers” METTL14, METTL3, RBM15, WTAP, ZC3H13, CBLL1, and “erasers” FTO, ALKBH5, all of which shared the key task of m6A methylation modification. The data were analyzed visually by ggplot2 software package.

### Correlations of SOCS3 Expression With 5mC Associated Genes in OLF

The R software package was utilized to evaluate the correlation between the expression of SOCS3 and the expression of 5mC regulators in OLF, including “writers” DNMT1, DNMT3A, and DNMT3B, “readers” MBD1, MBD2, MBD3, MBD4, MECP2, NEIL1, NTHL1, SMUG1, TDG, UHRF1, UHRF2, UNG, ZBTB38, ZBTB33, and ZBTB4, and “erasers” TET1, TET2, and TET3, all of which shared the key task of DNA methylation modification. The data were analyzed visually by ggplot2 software package.

### Clinical Specimens From Patients With Thoracic Ossification of Ligamentum Flavum and Healthy Individuals

The study protocol was approved by the Ethics Committee for Human Subjects of the Peking University Third Hospital in accordance with the Declaration of Helsinki (PUTH-REC-SOP-06-3.0-A27, #2014003). The experimental group included 10 adult patients diagnosed with TOLF who underwent surgical decompression, and the control group included 10 adult patients diagnosed with thoracic disc herniation or spine fracture from June 2020 to December 2020. Patients with ankylosing spondylitis (AS), diffuse idiopathic skeletal hyperostosis (DISH), rheumatoid arthritis, spinal tumors, spinal infections, and other systemic autoimmune diseases were excluded. Moreover, patients with hypertension, diabetes, and arteriosclerosis were also eliminated. The detailed patients’ clinical data are described in **Supplementary Table S1**, which showed there were no significant differences in age (60.50 ± 6.31 years vs. 60.80 ± 8.25 years, *P* = 0.928), gender, and BMI (27.76 ± 3.51 kg/m^2^ vs. 27.09 ± 3.55 kg/m^2^, *P* = 0.674) between the two groups.

### Validation of Candidate ORDEGs by qRT-PCR and Key 5mC/m6A Modifiers by Western Blotting

All candidate ORDEGs mRNA expression in the ligamentum flavum samples was determined by qRT-PCR. Total RNA was extracted with TRIzol reagent (Invitrogen Corporation, CA), and cDNAs were synthesized with a SuperScript III First-Strand Synthesis System for Reverse transcription (Invitrogen Corporation). The qRT-PCR for the mRNA level was carried out using SYBR Green I (TaKaRa, Tokyo, Japan) with the Bio-Rad iQ5 system (Bio-Rad, California). The relative gene expression levels were calculated using the 2^-ΔΔCt^ method. The primer sequences are listed in **Supplementary Table S2**. All experiments were performed in triplicate. The protein level of key 5mC/m6A modifiers was detected by Western blotting according to the standard experimental protocol. The primary antibodies included: FTO (CST; 1:1000), DNMT1 (CST; 1:1000), and SOCS3 (Abcam; 1:1000).

### Statistical Analysis and Visualization

All statistical analyses and visualization were performed using R software (version 3.6.3), SPSS 22.0 (IBM Analytics, United States), and GraphPad Prism version 8 (GraphPad Software, La Jolla, CA). Nonparametric test was used to examine the statistical relationship between two non-normally distributed data, and parametric test was used to examine the statistical relationship between two normally distributed data. Gene expression levels between two groups were analyzed by Student’s *t*-test. The expression of hub ORDEGs was compared for correlation analysis using the Pearson test. All data were expressed as mean ± standard deviation. *P* < 0.05 was considered as statistically significant.

## Results

### Identification of All ORDEGs in OLF

**Figure 1:**
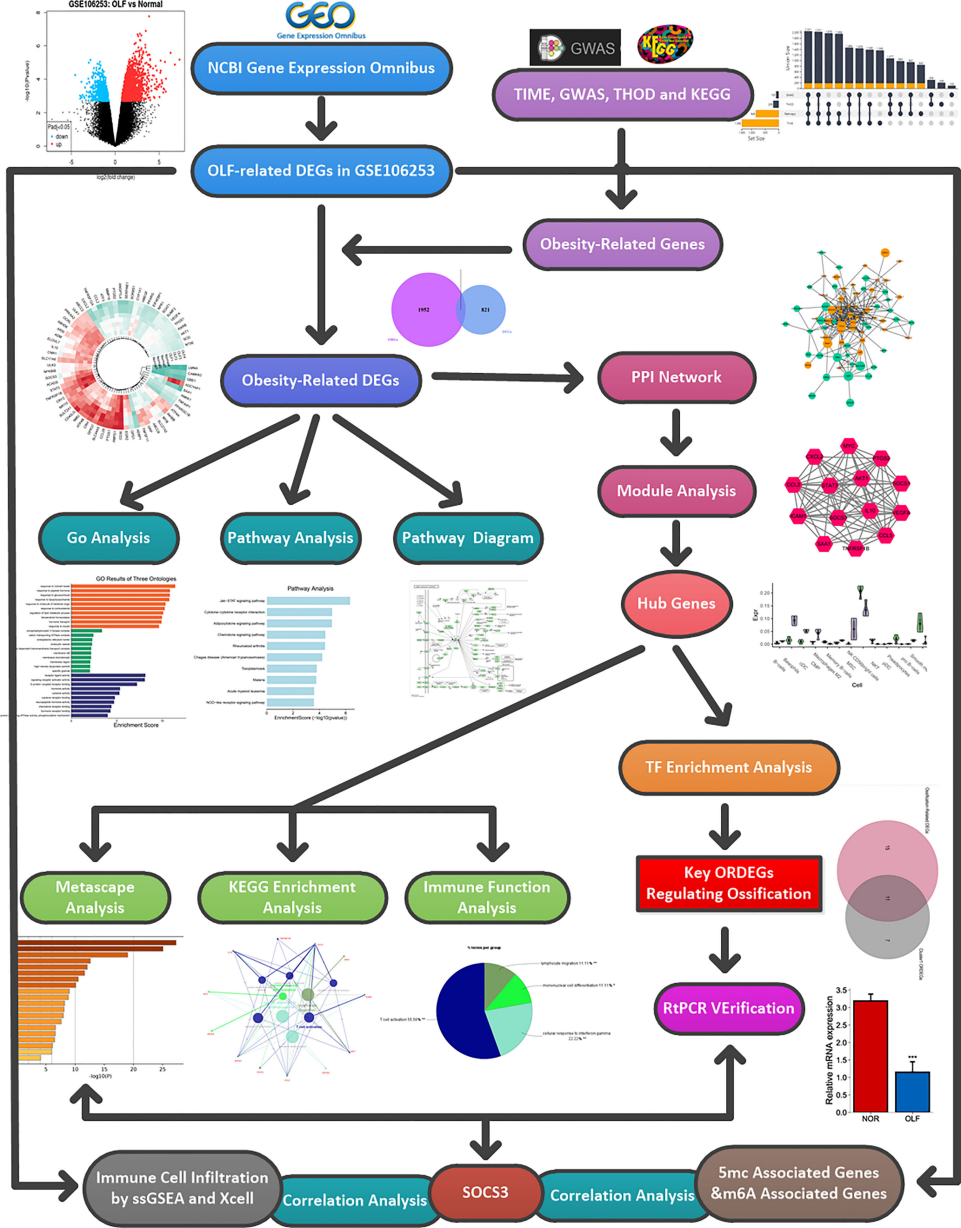
Flow chart of the whole analysis process.

The detailed workflow diagram of this study is depicted in **Figure 1**. After the raw data were processed by R software for background correction and data normalization (**Supplementary Figure S1A**), a total of 920 DEGs, consisting of 532 up-regulated genes and 388 down-regulated genes were identified between OLF samples and normal controls (**Supplementary Figure S1B**). The clustering heatmap showed that the top 40 DEGs could clearly distinguish OLF tissues from normal tissues (**Supplementary Figure S1C**). After deleting overlapping genes, a total of 2051 obesity-related genes in Homo sapiens were obtained from the above four databases (**Supplementary Figure S1D**). After intersection of these 920 DEGs and 2051 ORGs, 99 ORDEGs in OLF were ultimately identified (**Supplementary Figure S1E**). The two-dimensional PCA depicted a significant difference in these genes to allow further analysis (**Supplementary Figure S1F**), including 54 up-regulated genes and 55 down-regulated genes (**Supplementary Figure S1G**). The expression profile of the top 70 ORDEGs was intuitively visualized through a circular heatmap (**Supplementary Figure S1H**).

### GO Terms Enrichment Analysis of ORDEGs Pertaining Specific MF, BP, and CC

**Figure 2:**
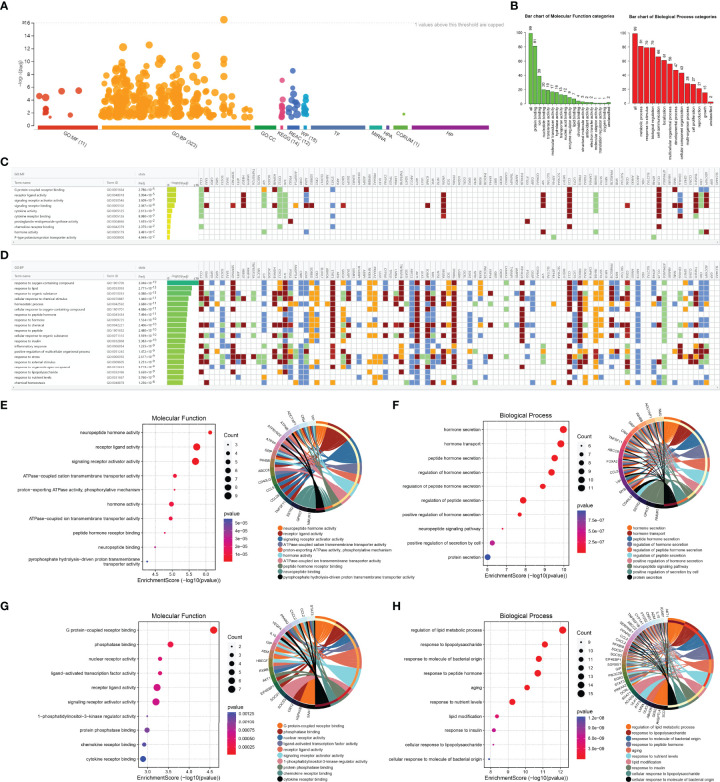
GO analyses of ORDEGs between OLF and control samples. **(A)** Manhattan plot showed a significant dysregulation in all categories, which included GO with 11 significant pathways for MF, 323 significant pathways for BP, 14 significant pathways for KEGG, 12 significant pathways for REAC, and 18 significant pathways for WP. **(B)** WebGestalt annotated the number of genes enriched in different pathways of MF and BP. **(C)** Profiler analysis indicated the GO terms analysis in MF. **(D)** Profiler analysis indicated the GO terms analysis in BP. **(E)** GO histogram plot and cnetplot show the enriched functions of up-regulated genes in MF. **(F)** GO histogram plot and cnetplot show the enriched functions of up-regulated genes in BP. **(G)** GO histogram plot and cnetplot show the enriched functions of down-regulated genes in MF. **(H)** GO histogram plot and cnetplot show the enriched functions of down-regulated genes in BP.

The ORDEGs in OLF were subjected to functional enrichment analysis using g: Profiler, and a significant dysregulation was observed in all categories analyzed, which included GO with 11 significant pathways for MF, 323 significant pathways for BP, 14 significant pathways for KEGG, 12 significant pathways for REAC, and 18 significant pathways for WP (**Figure 2A**). WebGestalt annotated the number of genes enriched in different pathways of MF and BP (**Figure 2B**). g: Profiler analysis indicated that the GO terms analysis in MF highlighted “G protein-coupled receptor binding”, “receptor ligand activity”, “signaling receptor activator activity”, “signaling receptor binding”, and “cytokine activity” (**Figure 2C**). For GO BP analysis, the top 20 are displayed in **Figure 2D**. Most pathways involved metabolic processes, such as “phosphorus metabolic process”, “lipid metabolic process”, “phosphate-containing compound metabolic process”, “positive regulation of cellular metabolic process”, and “monocarboxylic acid metabolic process”. Cellular responses were also represented, with the rest of the top significant terms pertaining to “response to oxygen-containing compound”, “response to lipid”, “response to organic substance”, “cellular response to chemical stimulus”, and “cellular response to oxygen-containing compound”. Furthermore, the biological functions of up-regulated and down-regulated genes were analyzed, separately. GO analysis identified that the up-regulated ORDEGs were significantly enriched in BP, including hormone secretion, hormone transport, and peptide hormone secretion (**Figure 2F**), whereas down-regulated ORDEGs were mainly enriched in regulation of lipid metabolic process, response to lipopolysaccharide, and response to molecule of bacterial origin (**Figure 2H**). Moreover, MF demonstrated that up-regulated genes were mainly related to neuropeptide hormone activity, receptor ligand activity and signaling receptor activator activity (**Figure 2E**), and down-regulated were mainly enriched in G protein-coupled receptor binding, phosphatase binding, and nuclear receptor activity (**Figure 2G**). These genes could be related to multiple biological pathways orchestrating OLF pathogenesis.

### Enrichment Pathway Analysis of ORDEGs Highlighted Specific Processes-Involvement

**Figure 3:**
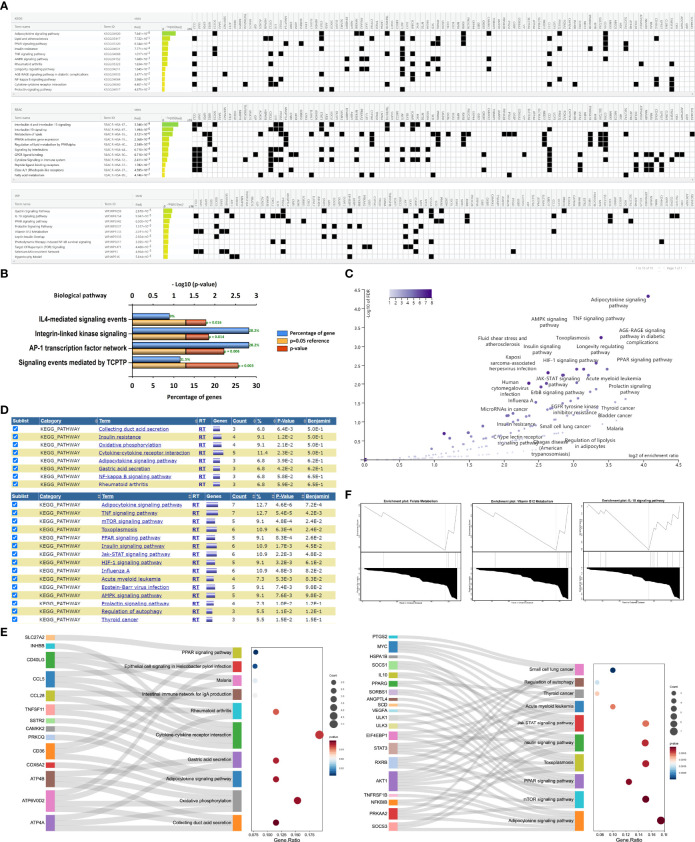
Pathway enrichment analyses of ORDEGs between OLF and control samples. **(A)** Profiler analysis showed the top 10 significant KEGG pathways, REAC pathways, and WP WikiPathways. **(B)** Funrich analysis indicated the 4 significant biological pathways. **(C)** Over-representation analysis of WebGestalt showed enrichment pathways of down-regulated genes, but no enriched results of up-regulated genes. **(D)** DIVID analysis showed enrichment pathways of down-regulated genes and up-regulated genes. **(E)** KEGG histogram plot showed the enriched pathways of up-regulated genes and down-regulated genes. **(F)** GSEA analysis showed significant enrichment pathways were involved in folate metabolism, vitamin 12 metabolism, and IL-18 signaling pathway in WP dataset.

All ORDEGs were subjected to pathway analysis in KEGG, REAC, and WP. g: Profiler analysis highlighted 15 significant KEGG pathways, 12 significant REAC pathways, and 18 WP WikiPathways. Their top 10 pathways, ranked by their significance, are reported in **Figure 3A**, respectively. Interestingly, inflammatory response, immune regulation, and lipid metabolism process seemed to be the most represented terms in all three datasets, such as TNF signaling, interleukins signaling, NF-kappa B signaling, prolactin signaling, adipocytokine signaling, and PPAR signaling. Moreover, the results of the enrichment analysis by Funrich indicated that the biological pathways were enriched in the signaling events mediated by TCPTP (T-cell protein tyrosine phosphatase), AP-1 transcription factor network, integrin-linked kinase signaling, and IL4-mediated signaling events, all of which were associated with inflammation or immune responses (**Figure 3B**). To further evaluate the distinctive functions of these genes, we applied WebGestalt, DIVID, and KEGG pathway analysis to analyze up-regulated and down-regulated genes, respectively. Over-representation analysis of WebGestalt showed down-regulated genes were enriched in adipocytokine signaling pathway, PPAR signaling pathway, TNF signaling pathway, and AMPK signaling pathway, but no enriched results were observed in up-regulated genes (**Figure 3C**). DIVID analysis demonstrated down-regulated genes were enriched in adipocytokine signaling pathway, TNF signaling pathway, mTOR signaling, PPAR signaling pathway, and JAK-STAT signaling pathway while up-regulated genes were enriched in collecting duct acid secretion, insulin resistance, and oxidative phosphorylation (**Figure 3D**). KEGG analysis revealed that the top 10 signaling pathways of the up-regulated and down-regulated ORDEGs (**Figure 3E**), respectively, and adipocytokine signaling pathway, mTOR signaling pathway, PPAR signaling pathway, and JAK-STAT signaling pathway were significantly activated in the gene sets. Furthermore, GSEA results revealed significant enrichment pathways were folate metabolism, vitamin 12 metabolism, and IL-18 signaling pathway in the WP dataset (**Figure 3F**). Taken together, the most significantly enriched pathways were the adipocytokine signaling (**Supplementary Figure S2A**), TNF signaling (**Supplementary Figure S2B**), AMPK signaling (**Supplementary Figure S2C**), and another two remarkable crosstalk pathways, mTOR signaling (**Supplementary Figure S2D**) and JAK-STAT signaling (**Supplementary Figure S2E**).

### PPI Network Construction and Module Analysis Identified the Leading Functional Network

**Figure 4:**
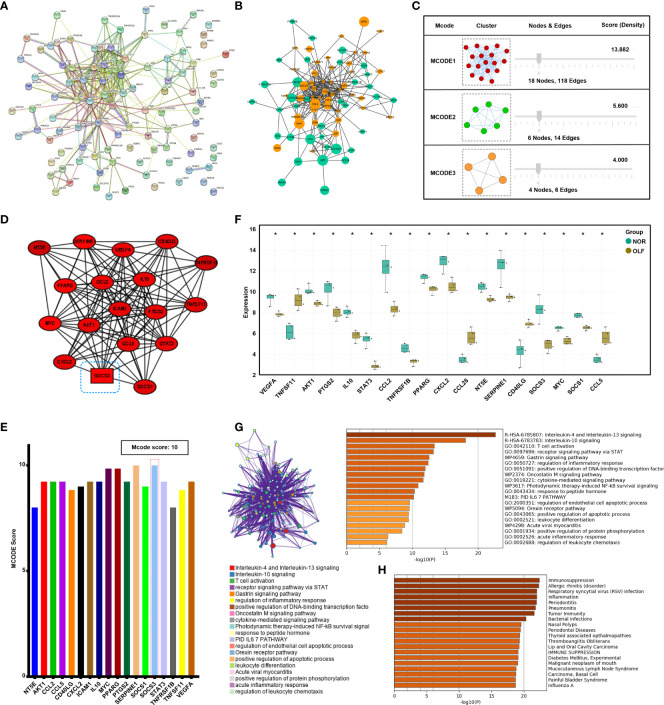
Protein-protein interaction network construction and modular analysis. **(A)** Construction of the PPI network based on all 99 ORDEGs in STRING 11.5. **(B)** The PPI network of these ORDEGs was displayed in the Cytoscape software. Up-regulated genes are marked in light green; down-regulated genes are marked in light orange. **(C)** Three significant modules from the PPI network were extracted by MCODE analysis. **(D)** The most highly connected sub-network (cluster rank 1; Score 13.882) identified by module analysis included 18 nodes and 118 interactions. **(E)** Connection scores of all genes in the leading cluster. **(F)** Box plot displayed the expression of all genes in the leading clusters; *p < 0.05. **(G)** Metascape functional annotation for hub genes in the leading cluster. **(H)** Enrichment analysis in DisGeNET for hub genes in the leading cluster.

By integrating ORDEG pairs with a combined score > 0.4, the PPI network was constructed after removing unconnected nodes (**Figure 4A**). Cytoscape visualized the PPI network of ORDEGs, which consisted of 81 nodes (DEGs) and 313 edges (**Figure 4B**), accounting for 81.82% of all DEGs. Considering the clustering coefficient, average shortest path length, betweenness centrality, and closeness centrality, the topology property of the PPI network was in power-law distribution (**Supplementary Figure S3**). Moreover, three significant modules from the PPI network were extracted by MCODE (**Figure 4C**). The most highly connected sub-network (cluster rank 1; Score 13.882) was obtained from the PPI network complex (**Figure 4D**), consisting of 18 nodes (AKT1, CCL2, CCL5, CD40LG, CXCL2, ICAM-1, IL10, MYC, NT5E, PPARG, PTGS2, SERPINE1, SOCS1, SOCS3, STAT3, TNFRSF1B, TNFSF11, VEGFA) and 118 interactions, which was considered a critical functional module. SOCS3 was the seed gene with the highest MCODE score in this network (**Figure 4E**). **Supplementary Table S3** shows the detailed information and molecular functions of the key genes. Box plot displayed the expression of all genes in three clusters (**Figure 4F**). Furthermore, Metascape results revealed that these hub genes were mainly enriched in inflammatory/immune related responses (**Figure 4G**), such as interleukin-4 and interleukin-13 signaling, interleukin-10 signaling, and T cell activation, and enrichment analysis in DisGeNET also showed immunosuppression and inflammation in OLF (**Figure 4H**).

### Functional Annotations of hub ORDEGs Revealed Specific Inflammatory Signaling and Immune Responses in OLF

**Figure 5:**
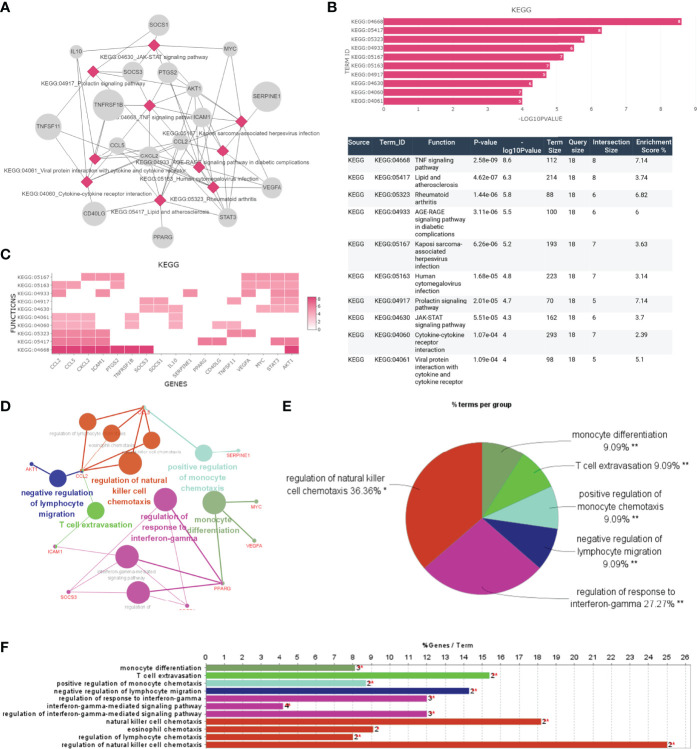
The function analysis of hub genes. **(A–C)** The most significant pathway and related genes. The results show that these hub genes are mainly involved in TNF signaling pathway, prolactin signaling pathway, JAK-STAT signaling pathway, and cytokine-cytokine receptor interaction. **(D–F)** The most significant immune response and related genes. The results show that these hub genes are mainly involved in regulation of natural killer cell chemotaxis, regulation of response to interferon-gamma. *p < 0.05, **p < 0.01.

Furthermore, we focused on the analysis of the significant inflammatory pathways and immune processes of these hub genes. KEGG results indicated that these genes were related to TNF signaling pathway and JAK-STAT signaling pathway, which were universally acknowledged as important inflammation-related pathways (**Figures 5A, B**). AKT1, CCL2, CCL5, CXCL2, ICAM-1, PTGS2, SOCS3, and TNFRSF1B were enriched in TNF signaling while SOCS1, SOCS3, AKT1, IL-10, MYC, and PTGS2 were involved in JAK-STAT signaling (**Figure 5C**). Further subgroup analysis was conducted to separately investigate possible immune functions of these genes, and regulation of natural killer cell chemotaxis (36.36%), regulation of response to interferon-gamma (27.27%), negative regulation of lymphocyte migration (9.09%) and positive regulation of monocyte cell chemotaxis (9.09%), T cell extravasation (9.09%), and monocyte differentiation (9.09%) were identified as the potential immune responses involved (**Figures 5D-F**).

### TFEA and qRT-PCR Validation of Hub ORDEGs Involved in Regulation of Ossification

**Figure 6:**
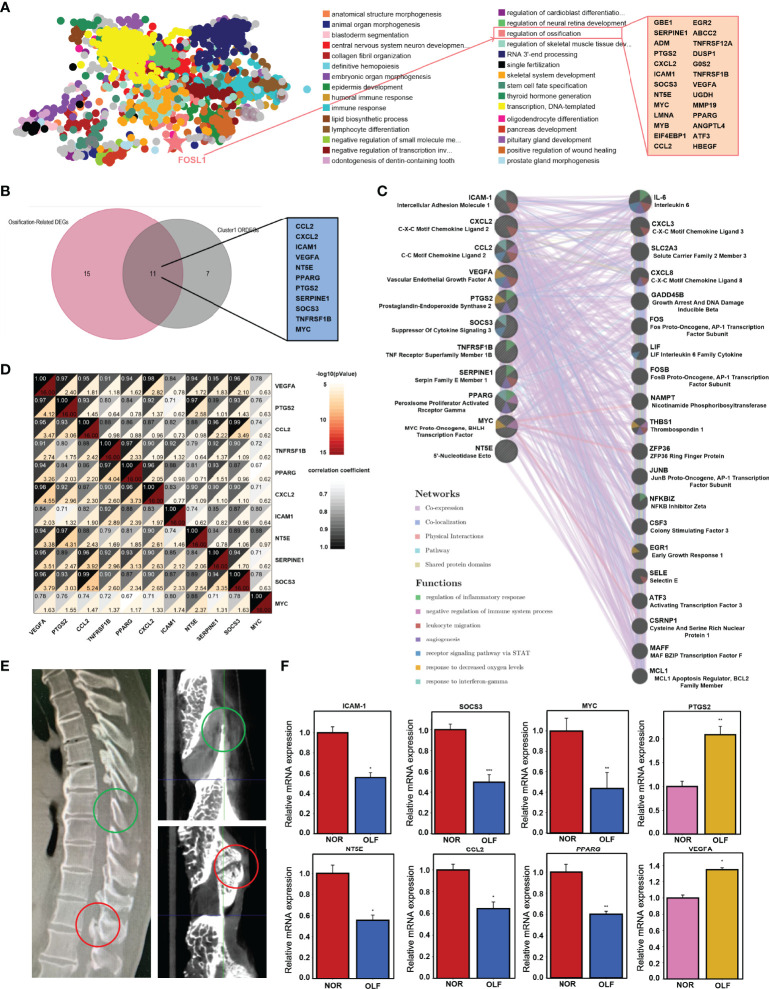
TF enrichment analysis and qRT-PCR validation of hub ORDEGs. **(A)** TF enrichment analysis by ChEA3 identified 28 genes with function of regulating ossification. **(B)** Venn diagram showed the intersection between 28 genes and the leading cluster genes to obtain the 11 candidate ORDEGs. **(C)** Candidate ORDEGs and their co-expression genes were analyzed using GeneMANIA. **(D)** Correlation analysis of 11 candidate ORDEGs. **(E)** Sagittal CT imaging of one TOLF patient and micro-CT views of its clinical specimen. **(F)** Validation of the expression of these genes by qRT-PCR. The expression levels of CCL2, ICAM-1, MYC, NT5E, PPARG, PTGS2, and SOCS3 were down-regulated, which were paralleled with microarray results, but PTGS2 and VEGFA were up-regulated. *p < 0.05, **p < 0.01, ***p < 0.001.

To further screen genes with the function of regulation of ossification, TFEA by ChEA3 identified 28 genes (GBE1, SERPINE1, ADM, PTGS2, CXCL2, etc.) (**Figure 6A**). Then, these 28 genes and hub ORDEGs were intersected to obtain the 11 candidate ORDEGs which had the potential of regulating ligamentum flavum ossification, including CCL2, CXCL2, ICAM-1, MYC, NT5E, PPARG, PTGS2, SERPINE1, SOCS3, TNFRSF1B, and VEGFA (**Figure 6B**). Furthermore, co-expression analysis based on GeneMANIA database showed a complex PPI network with co-expression of 65.62%, genetic interactions of 15.03%, physical interactions of 8.28%, shared protein domains of 3.99%, pathway of 3.86%, and co-localization of 3.21%, whose functions were mainly associated with regulation of inflammatory response and immune process (**Figure 6C**). Correlation analysis among 11 genes was conducted to investigate their whole interrelations (**Figure 6D**). Among them, SOCS3 and CCL2 had the highest positive correlation with a person’s correlation coefficient of 0.99. To confirm the accuracy of the prediction results, qRT-PCR was used to detect the expression of the above 11 candidate ORDEGs based on clinical specimens (**Figure 6E**). Eventually, except for CXCL2 and SERPINE1, changes in the expression levels of CCL2, ICAM-1, MYC, NT5E, PPARG, PTGS2, and SOCS3 were paralleled in microarray and qRT-PCR, but there was an opposite trend in PTGS2 and VEGFA expression (**Figure 6F**).

### Identification of Immune Infiltrating Landscape and Differential Immune Cell Types in OLF

**Figure 7:**
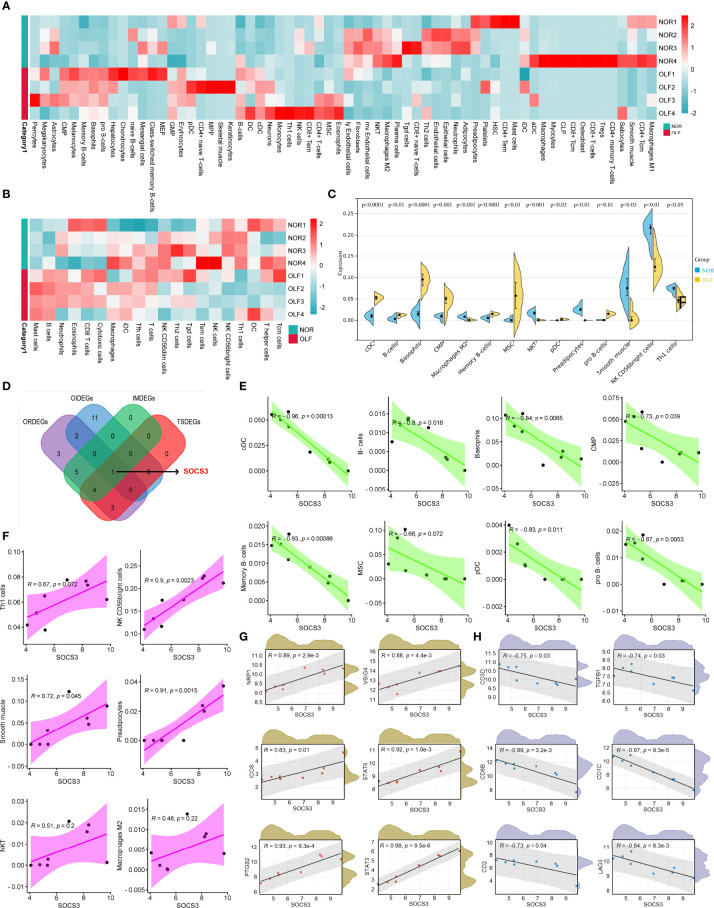
Correlation analysis of SOCS3 and OIICs as well as immune marker sets. **(A)** Heatmap of 64 infiltrating immune cells between normal and OLF tissues. **(B)** Heatmap of 23 infiltrating immune cells between normal and OLF tissues. **(C)** The expression level of 14 differential immune cells between normal and OLF samples. **(D)** Venn diagram showed the intersection among ORDEGs, OIDEGs, IMDEGs, and TSDEGs to identify SOCS3 as the core gene. **(E)** SOCS3 expression showed a negative correlation with the levels of cDC cells, B-cells, basophils, CMP, memory B-cells, MSC, pDC, and pro B-cells. **(F)** SOCS3 showed a positive correlation with the levels of Th1 cells, NK CD56 bright cells, smooth muscle cells, preadipocytes, NKT cells, and macrophages M2. **(G)** The expression of SOCS3 was positively correlated with the immune markers NRP1, VSIG4, PTGS2, ICOS, STAT3 and STAT6. **(H)** The expression of SOCS3 was negatively correlated with the immune markers CD1C, CD3D, CD2, CD8B, TGFB1 and LAG3.

The above results demonstrated that the hub genes were highly enriched in immune-related or inflammation-related responses and pathways. Therefore, combining ssGSEA and xCell algorithms comprehensively evaluated infiltrating immune cell types in each sample based on the gene expression matrix from the GSE106253 (**Figures 7A, B**). Ultimately, 14 types of OIICs were significantly altered, including natural killer T (NKT) cells, macrophages M2, NK CD56 bright cells, common myeloid progenitor (CMP) cells, basophils, conventional dendritic cells (cDCs), plasmacytoid dendritic cells (pDCs), pro B-cells, memory B cells, B cells, T helper 1 (Th1) cells, mesenchymal stem cells (MSC), smooth muscle cells, and preadipocytes (**Figure 7C**).

### Correlation Analysis Between SOCS3 Expression and OIICs as Well as Immune Marker Sets

Our previous research has identified 10 OLF-related and immune-related differentially expressed genes (OIDEGs) (20). Eleven ORDEGs, OIDEGs, TNF signaling-enriched DEGs (TSDEGs), and immune response-enriched DEGs (IMDEGs) were overlapped to obtain SOCS3 as the core gene (**Figure 7D**). We further analyzed correlation between SOCS3 expression and OIICs in OLF. As shown in **Figure 7E**, SOCS3 expression showed a negative correlation with the levels of cDC cells (R = -0.96), B-cells (R = -0.8), basophils (R = -0.84), CMP (R = -0.73), memory B-cells (R = -0.93), MSC (R = -0.66), pDC (R = -0.83), and pro B-cells (R = -0.87). Besides, SOCS3 had a positive correlation with the infiltration level of Th1 cell (R = 0.67), NK CD56 bright cells (R = 0.90), smooth muscle cells (R = 0.72), preadipocytes (R = 0.91), NKT cells (R = 0.51), and macrophages M2 (R = 0.48) (**Figure 7F**). Furthermore, we analyzed the correlation between SOCS3 and immune markers of various immune cells in OLF (**Supplementary Table S4**). As shown in **Figures 7G, H**, the expression of SOCS3 was significantly correlated with the immune markers NRP1 (R = 0.89) and CD1C (R = 0.92) of DC cells. Moreover, SOCS3 expression was significantly correlated with VSIG4 (R = 0.88) of M2 macrophage and PTGS2 (R = 0.93) of M1 macrophage, which indicated that SOCS3 might regulate macrophage polarization in OLF. We also found that the expression level of SOCS3 was significantly correlated with most of the immune markers of different T cells in OLF, including CD3D (R = -0.75) and CD2 (R = -0.73) of T cells, CD8B (R = -0.93) of CD8^+^ T cells, TGFB1 (R = -0.74) of Tregs, LAG3 (R = -0.84) of T cell exhaustion, and STAT6 (R = 0.92) of Th2 cells. These findings implied that SOCS3 might be involved in the T cell immune response in OLF.

### SOCS3 Expression May Be Associated With m6A RNA Methylation Regulators in OLF

**Figure 8:**
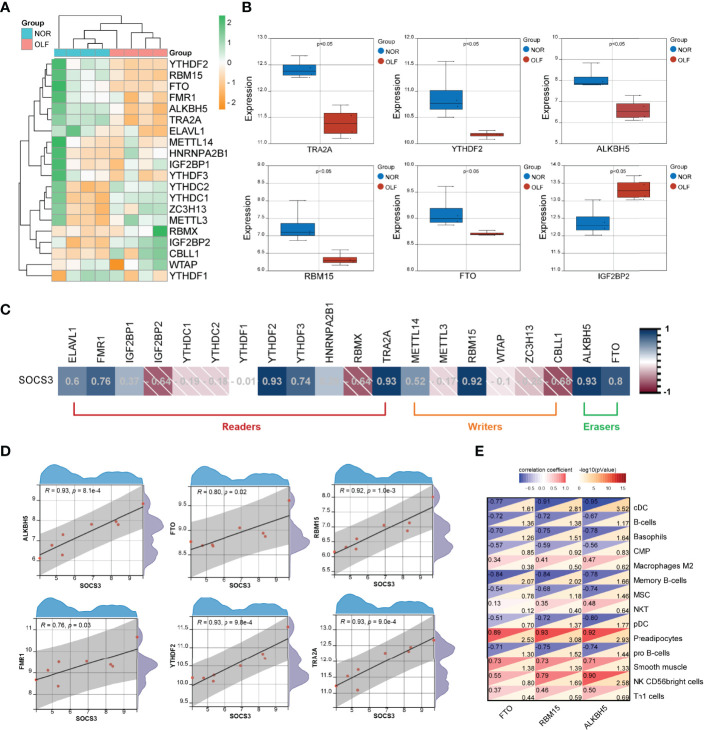
Correlation analysis of SOCS3 and m6A RNA methylation regulators in OLF. **(A)** Heatmap of 20 m6A regulators in OLF. **(B)** Box plot showing six differentially expressed m6A regulators (TRA2A, YTHDF2, RBM15, IGF2BP2, FTO, and ALKBH5) between OLF and control tissues. **(C)** Correlation heatmap between SOCS3 expression and 20 m6A regulators. **(D)** SOCS3 expression was significantly positively correlated with 6 m6A-related genes, including FTO, YTHDF2, RBM15, FMR1, ALKBH5, and TRA2A. **(E)** Correlation heatmap between these three m6A modifiers (FTO, RBM15, and ALKBH5) and OIICs.

m6A modification has been identified to affect RNA metabolism, such as stability, translation, degradation, transport, and splicing. Therefore, we tried to analyze whether SOCS3 expression was related to m6A regulators in OLF. Based on GSE106253 dataset, the expression pattern of 20 m6A regulators in OLF is presented in **Figure 8A**. The results revealed that 6 m6A regulators (TRA2A, YTHDF2, RBM15, IGF2BP2, FTO, and ALKBH5) were significantly altered between OLF and control samples (**Figure 8B**). Then, we constructed the correlation analysis between the expression of SOCS3 and 20 m6A-related genes (**Figure 8C**), and we found SOCS3 expression was significantly positively correlated with 6 m6A-related genes, including FTO (R = 0.8), YTHDF2 (R = 0.93), RBM15 (R = 0.92), FMR1 (R = 0.76), ALKBH5 (R =0.93), and TRA2A (R = 0.93) (**Figure 8D**). Notably, FTO and ALKBH5 were erasers, and RBM15 was one type of writer, which could play crucial post-transcriptional regulatory roles. Accumulating evidence showed m6A played an essential role in regulating both innate and adaptive immune cells through various mechanisms. Therefore, we further investigated the correlation between these three m6A modifiers and OIICs. As shown in **Figure 8E**, FTO, ALKBH5, and RBM15 were significantly related to cDCs, preadipocytes, and memory B cells.

### SOCS3 Expression and m6A RNA Methylation May Be Associated With DNA Methylation Regulators in OLF

**Figure 9:**
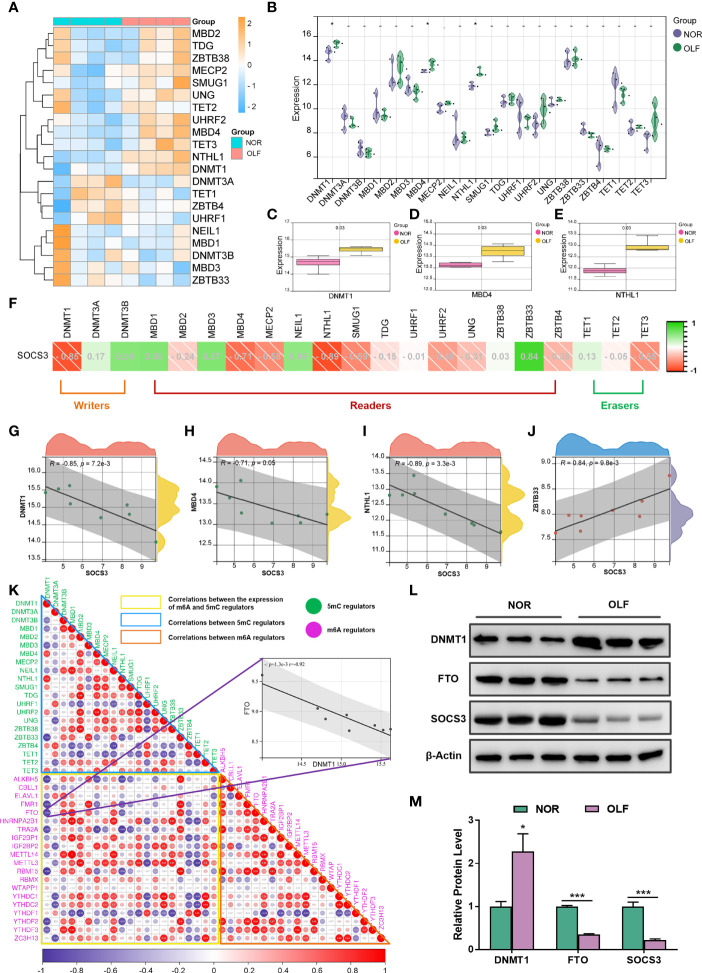
Correlation analysis of SOCS3 and 5mC regulators in OLF. **(A)** Heatmap of 21 5mC regulators in OLF. **(B)** Volcano plot showing the expression of 21 5mC regulators in OLF. **(C)** Box plot showing three differentially up-regulated DNMT1 in OLF. **(D)** Box plot showing three differentially up-regulated MBD4 in OLF. **(E)** Box plot showing three differentially up-regulated NTHL1 in OLF. **(F)** Correlation heatmap between SOCS3 expression and 21 5mC regulators. **(G)** SOCS3 expression was significantly negatively correlated with DNMT1. **(H)** SOCS3 expression was significantly negatively correlated with MBD4. **(I)** SOCS3 expression was significantly negatively correlated with NTHL1. **(J)** SOCS3 expression was significantly positively correlated with ZBTB33. **(K)** Correlation heatmap between the expression of m6A and 5mC regulators, and DNMT1 had a strong negative correlation with FTO. **(L, M)** The protein level of FTO and SOCS3 were significantly down-regulated while the protein level of DNMT1 was significantly up-regulated in OLF samples compared to controls. *p < 0.05, ***p < 0.001.

Accumulating evidence suggests that DNA methylation alterations play a significant role in osteogenesis and inflammation, and our recent findings demonstrated an altered genome-wide DNA methylation profile in TOLF (17). Therefore, we tried to analyze crosstalk between m6A and 5mC regulators on SOCS3 expression in OLF. First, we evaluated the expression of 21 5mC-related genes in OLF (**Figures 9A, B**), and found the expression of DNMT1, MBD4, and NTHL1 were differentially up-regulated (**Figures 9C–E**). Then, we detected the correlation analysis between the expression of SOCS3 and 21 5mC-related genes (**Figure 9F**), and we found SOCS3 expression was significantly correlated with DNMT1 (R = 0.85, **Figure 9G**), MBD4 (R = -0.71, **Figure 9H**), NTHL1 (R = -0.89, **Figure 9I**), and ZBTB33 (R = 0.84, **Figure 9J**). Furthermore, we investigated the correlations between the expression of m6A and 5mC regulators, and our results showed correlated expression patterns for genes within the same regulator class and even high correlations between the expression of m6A and 5mC regulators (**Figure 9K**). DNMT1, as a writer in DNA methylation, has significantly increased expression and a strong negative correlation with FTO (R = -0.92), which could be considered as a crucial 5mC regulator in OLF.

### Validation of DNMT1, FTO, SOCS3 Protein Level in OLF by Western Blotting

When taking mRNA expression level and functional characteristics of 5mC/m6A-related genes as well as correlation analysis results into account, we inferred crosstalk between DNMT1 and FTO might play an important part in affecting SOCS3 expression and regulatory mechanism in OLF. To validate their expression changes, we examined the protein level of DNMT1, FTO, and SOCS3 in ligamentum flavum tissues from TOLF patients and normal controls. As shown in **Figures 9L, M**, the protein level of FTO and SOCS3 were significantly down-regulated while the protein level of DNMT1 was significantly up-regulated in OLF samples compared to controls, which were similar to the results of mRNA expression from microarray data.

## Discussion

As a multifactorial disease, obesity has been considered as an important risk factor associated with the pathogenesis of OLF (7, 8). However, obesity-mediated genetic mechanism underlying OLF has not been fully understood. In this study, we integrated bioinformatics algorithms to comprehensively decipher obesity-related gene signatures and verified 11 key ORDEGs with the function of regulating ossification by qRT-PCR. Furthermore, down-regulated SOCS3 was identified as a core gene in OLF, and we found the function of SOCS3 was mainly correlated with inflammatory signaling pathways (TNF signaling pathway, JAK-STAT signaling pathway, etc.) and multiple immune infiltrates (dendritic cells, macrophage, T cells, etc.), and its expression alterations might be related to 5mC/m6A modifiers. Finally, reduced SOCS3 and FTO expression and increased DNMT1 level in OLF were validated by Western blotting. Collectively, the current study elaborated the expression profile and functional characteristics of obesity-related genes in OLF and revealed SOCS3 associated with immune infiltrates and 5mC/m6A modifiers for the first time, which might provide novel insights into the pathogenesis and treatment strategies of obesity-related OLF.

The GO and pathway enrichment analysis are of significant importance for appreciating the whole biological functions and molecular mechanisms of these ORDEGs profiles in OLF. GO analysis showed that all ORDEGs were mainly enriched in metabolic responses and obesity status. Besides, up-regulated and down-regulated ORDEGs were found to be enriched in inflammation-related pathways, such as adipocytokine signaling pathway and TNF-α signaling pathway, which were implicated in several obesity-related biological reactions. Our previous study has proven that leptin/LepR signaling, an important adipocytokine signaling, could promote osteogenesis differentiation through STAT3 signaling pathway, which indicated the intrinsic interaction between obesity and OLF (10). Moreover, our proteomics coupling with experimental verification also elucidated the definite role and mechanisms of TNF-α in development of OLF (21, 22). Besides JAK-STAT signaling, we also identified a potential functional pathway, mTOR signaling pathway. On one hand, numerous studies have demonstrated that mTOR activation was implicated in metabolic diseases, such as obesity and diabetes (23, 24). On the other hand, increasing evidence showed mTOR signaling could mediate the chondrogenesis, osteogenesis, and heterotopic ossification processes (25, 26). Therefore, it was speculated that mTOR signaling crosstalk might be a potential molecular mechanism linking obesity and OLF, which deserves further investigation.

PPI network construction and MCODE analysis can group and organize all the genes encoding proteins to screen hub genes and regulatory modules in the disease pathogenesis. By this means, one important gene cluster containing 18 hub genes was obtained. First, several cytokines or chemokines associated with inflammation and immunity were observed including CCL2, CCL5, CXCL2, IL-10, PTGS2, and TNFRSF1B. For instance, Luis et al. revealed that CCL2 and CCL5 participated in the immunomodulation of osteoblast differentiation during M1/M2 transition (27). Aimalie et al. found marrow adipocyte-derived CXCL2 in obese populations contributed to osteolysis (28). Moreover, STAT3 has been verified to play a significant role in the osteogenesis differentiation of ligamentum flavum cell (10). Importantly, suppressors of cytokine signaling (SOCS) family proteins form part of a classical negative feedback system that regulates cytokine signal transduction (29). SOCS1 and SOCS3 are involved in negative regulation of multiple cytokines signaling through the JAK-STAT pathway, thus they might become potential targets for progression of OLF (30). AKT1 can regulate many processes including metabolism, cell proliferation, growth, and angiogenesis, and has been proved to participate in terminal stages of endochondral bone formation that is the pathological nature of OLF (31). ICAM-1 has been considered as an important adhesion molecule involved in bone homeostasis (32). Tanaka et al. found that ICAM-1^+^ osteoblasts could bias bone turnover to bone resorption, which prompted some novel thought about whether ICAM-1 might be a potential therapeutic target for OLF (33). Yang et al. indicated that angiogenesis was responsible for development of OLF (34). VEGFA and MYC, as important participators and regulators in the angiogenesis process, might be associated with advancement of OLF (35, 36). Based on the Metascape database and ClueGO results, we found that these hub genes were mainly involved in biological processes related to inflammation and immunity, inflammatory pathways including TNF and JAK-STAT signaling, and immune responses such as regulation of interferon-gamma-mediated signaling and T cell extravasation. These results indicated that these hub genes played a vital role in inflammatory and immune response associated with obesity status in OLF. Afterward, based on TFEA and qRT-PCR verification, we confirmed that SOCS3, ICAM-1, NT5E, MYC, CCL2, and PPARG were potential ORDEGs that linked obesity and OLF pathogenesis.

SOCS proteins function as feedback inhibitors of the JAK-STAT signaling pathway, and they can terminate innate and adaptive immune responses (37, 38). In this study, we found SOCS3 was not only the seed gene in the key network, but also was only one immune-related gene, which was simultaneously enriched in TNF signaling, JAK-STAT signaling, and regulation of response to interferon-γ. To explore potential immunoregulatory functions of SOCS3 in OLF, 14 types of differential infiltrating immune cells in OLF were identified, and the correlation between SOCS3 and immune infiltration in OLF was investigated. We found that SOCS3 expression was significantly correlated with cDC cells, memory B-cells, NK CD56 bright cells, and preadipocytes. Moreover, the correlation between SOCS3 expression and immune cell marker genes revealed that SOCS3 might play a significant role in regulating OLF immunity, including DC cells (NRP1, CD1C), M1 macrophage (PTGS2), M2 macrophage (VSIG4), and various types of T cell. Previous research showed that SOCS3 deficiency promotes M1 macrophage polarization and inflammation (39). In addition, SOCS3 ablation could enhance DC-derived Th17 immune response by activating IL-6/STAT3 (40). These findings indicated that abnormally deficient SOCS3 might function in the regulation and recruitment of immune infiltrating cells, and thereby induce inflammation involved in OLF. However, rigorous experimental research is needed to explain the relationship between SOCS3 and specific immune responses in OLF.

m6A methylation has been documented to be the most pervasive internal mRNA modification in eukaryotes, which plays important roles in affecting RNA metabolism including RNA splicing, translation, stability, and translocation (41). Numerous studies demonstrated that regulators of m6A RNA methylation were involved in bone homeostasis and human osteogenesis diseases (42–44). More importantly, dysregulation of m6A has also been associated with the initiation and progression of spinal ligament ossification disorders. Yuan et al. found that the key m6A methyltransferase METTL3 could enhance ossification of the posterior longitudinal ligament *via* the lncRNA XIST/miR-302a-3p/USP8 axis (45). Wang et al. showed that m6A demethylase ALKBH5 promoted the osteogenesis of ligamentum flavum cells through BMP2 demethylation and AKT activation (18). Besides, accumulating evidence confirmed m6A participated in the pathogenesis of immune-related diseases by regulating both innate and adaptive immune cells through various mechanisms (46). In this study, we tried to probe whether low expression of SOCS3 was related to abnormal m6A modification in OLF. Results showed that SOCS3 expression was highly correlated with FTO, YTHDF2, RBM15, FMR1, ALKBH5, and IGF2BP2. Among them, FTO was first identified as a gene related to obesity and energy metabolism and was then recognized as an RNA m6A demethylase (47, 48). Analogously, we found FTO was down-regulated in OLF samples, which was positively correlated with SOCS3 expression, and it suggested that FTO degradation might increase SOCS3 stability and reduce SOCS3 expression in a specific m6A-dependent manner in OLF progression. Nevertheless, further research should be adopted to verify this hypothesis or whether several other m6A regulators also participate in the methylation of SOCS3.

Epigenetic mechanisms are usually envisioned as both sensors of cell exposure and effectors for reprograming cell fate, thus mediating cells toward certain transformed states (49, 50). Aberrant DNA hypermethylation at CpG islands leads to the transcriptional silencing (51). There are multiple factors accounting for DNA hypermethylation, which presents increased activity in gene promoters of DNMT1, DNMT3A, and DNMT3B, or reduced activity of TET1, TET2, and TET3 (52). In the present study, we only found that the expression of DNMT1 was significantly increased in OLF samples compared to controls, suggesting that DNMT1 hyperactivation might institute a major mechanism inducing genome-wide DNA hypermethylation in OLF. Moreover, we found not only was the expression of SOCS3 and DNMT1 negatively correlated, but the expression of DNMT1 and FTO was also negatively correlated, which revealed several possible mechanisms, that is DNMT1 deregulation of SOCS3 axis, DNMT1-methylated FTO-mediated m6A of SOCS3 axis, or both. Tao et al. found DNMT1 silencing of SOCS3 axis as a driver of cardiac fibroblast activation in diabetic cardiac fibrosis (53). Yu et al. found DNA methylation of FTO could promote renal inflammation by enhancing m6A of PPAR-α in alcohol-induced kidney injury (54). Therefore, we deduced the underlying pathomechanism that the obese people were associated with the onset and progression of OLF. On one hand, persistent external factors, such as obesity-related systemic inflammation, induce DNA hypermethylation, and in turn, genome-wide repression of target genes including m6A modifiers and SOCS3. On the other hand, reduced activity of m6A modifiers alters SOCS3 mRNA levels in m6A-dependent manner in LF. Ultimately, the crosstalk between 5mC and m6A methylation converges to cause enhanced inflammation and abnormal ossification progression.

There are still some limitations in this study. Considering that OLF is an exceedingly rare disease that it is difficult to obtain sufficient clinical samples, we are regrettably unable to verify underlying interactions and molecular mechanisms of these hub genes at this stage. In the future, more in-depth experimental research will be carried out based on this article. Moreover, the exact mechanisms of immune reactions induced by SOCS3 need to be further investigated. In addition, the correlation between these key genes and clinical parameters should be studied in the future, which is important for clinical diagnosis and treatment. On all accounts, we have applied scientific bioinformatics algorithms and real-data validation to decipher these data and show new findings, which, to some extent, could be enlightening for the subsequent mechanism studies.

## Conclusion

To our best knowledge, this is the first study depicting distinct obesity-related genes profiles between OLF and normal controls based on transcriptome data mining, and 8 hub ORDEGs were ultimately identified by bioinformatics algorithms and experimental validation. The comprehensive functional annotations indicated these genes might mediate a detailed immune or inflammatory response or signaling pathways in the OLF pathogenesis. SOCS3 was identified as a core gene, which was associated with multiple immune infiltrates and 5mC/m6A modifiers in OLF, and the crosstalk between DNMT1 and FTO on affecting SOCS3 expression might play a significant role in OLF pathogenesis. Our investigations will provide new insights into obesity-related genes, epigenetic regulation, and immune-related mechanisms in OLF and pave new ways for related therapeutic targets.

## Data Availability Statement

Publicly available datasets were analyzed in this study. This data can be found here: https://www.ncbi.nlm.nih.gov/geo/query/acc.cgi?acc=GSE106253.

## Ethics Statement

The study protocol was approved by the Ethics Committee for Human Subjects of the Peking University Third Hospital in accordance with the Declaration of Helsinki (PUTH-REC-SOP-06-3.0-A27, #2014003). The patients/participants provided their written informed consent to participate in this study. Written informed consent was obtained from the individual(s) for the publication of any potentially identifiable images or data included in this article.

## Author Contributions

ZC and DF supported the research. BZ and LY conceived the original idea and the structure of the manuscript. BZ performed the experiments and drafted the first version of the manuscript. GC, XC, and TF assisted in the experiments and manuscript. XY collected the clinical samples. CS and LY provided critical feedback and helped revise the manuscript. All authors contributed to the article and approved the submitted version.

## Funding

This work was supported by National Natural Science Foundation of China (82072479, 81772381).

## Conflict of Interest

The authors declare that the research was conducted in the absence of any commercial or financial relationships that could be construed as a potential conflict of interest.

## Publisher’s Note

All claims expressed in this article are solely those of the authors and do not necessarily represent those of their affiliated organizations, or those of the publisher, the editors and the reviewers. Any product that may be evaluated in this article, or claim that may be made by its manufacturer, is not guaranteed or endorsed by the publisher.

## Abbreviations

OLF, ossification of ligamentum flavum; OLFCs, ossified ligamentum flavum cells; NLFCs, normal ligamentum flavum cells; ORDEGs, OLF-related and obesity-related differentially expressed genes; OIICs, OLF-related infiltrating immune cells; HO, heterotopic ossification; LF, ligamentum flavum; GEO, Gene Expression Omnibus; PPI, protein-protein interactions; ssGSEA, single sample gene set enrichment analysis; DEGs, differentially expressed genes; GO, gene ontology; BP, biology process; CC, cell component; MF, molecular function; KEGG, Kyoto Encyclopedia of Genes and Genomes; OPLL, ossification of posterior longitudinal ligament; TF, transcription factor; TFEA, TF enrichment analysis; REAC, Reactome; WP, WikiPathways; GSEA, Gene set enrichment analysis; AS, ankylosing spondylitis; DISH, diffuse idiopathic skeletal hyperostosis; SOCS, suppressors of cytokine signaling.
