# Supplementary material for: Deciphering Obesity-Related Gene Clusters Unearths SOCS3 Immune Infiltrates and 5mC/m6A Modifiers in Ossification of Ligamentum Flavum Pathogenesis
Source: Front Endocrinol (Lausanne). 2022 May 30;13:861567. doi: 10.3389/fendo.2022.861567 (PMC9196192; doi:10.3389/fendo.2022.861567)
Supplement: Supplementary file 1 [file DataSheet_1.docx]

Supplementary Material

# Supplementary Figures and Tables

## Supplementary Figures

**Supplementary Figure 1.** Identification and analysis of obesity-related DEGs in OLF. (A) The raw data was processed by R software for background correction and data normalization. (B) Volcano map of 920 DEGS, including 532 up-regulated genes and 388 down-regulated genes. Magenta dots represent genes with |logFC| > 1 and *P* < 0.05. (C) The two-way hierarchical clustering heatmap of top 40 genes. Each column in the heatmap represents a sample, and each row represents a gene. (D) A total of 2051 obesity-related gene lists in Homo sapiens were obtained from Integratomics TIME, GWAS, T-HOD and KEGG PATHWAY databases. (E) Venn diagram indicates the overlap of DEGs in OLF and obesity-related genes to obtain 99 ORDEGs. (F) The two-dimensional PCA depicted a significant difference in 99 ORDEGs. (G) Stem-and-Leaf Plot showed the expression change of 99 ORDEGs, including 54 up-regulated genes and 55 down-regulated genes. Horizontal axes represent genes, and vertical axes represents fold change. (H) Circular heatmap showed the expression profile of top 70 ORDEGs.


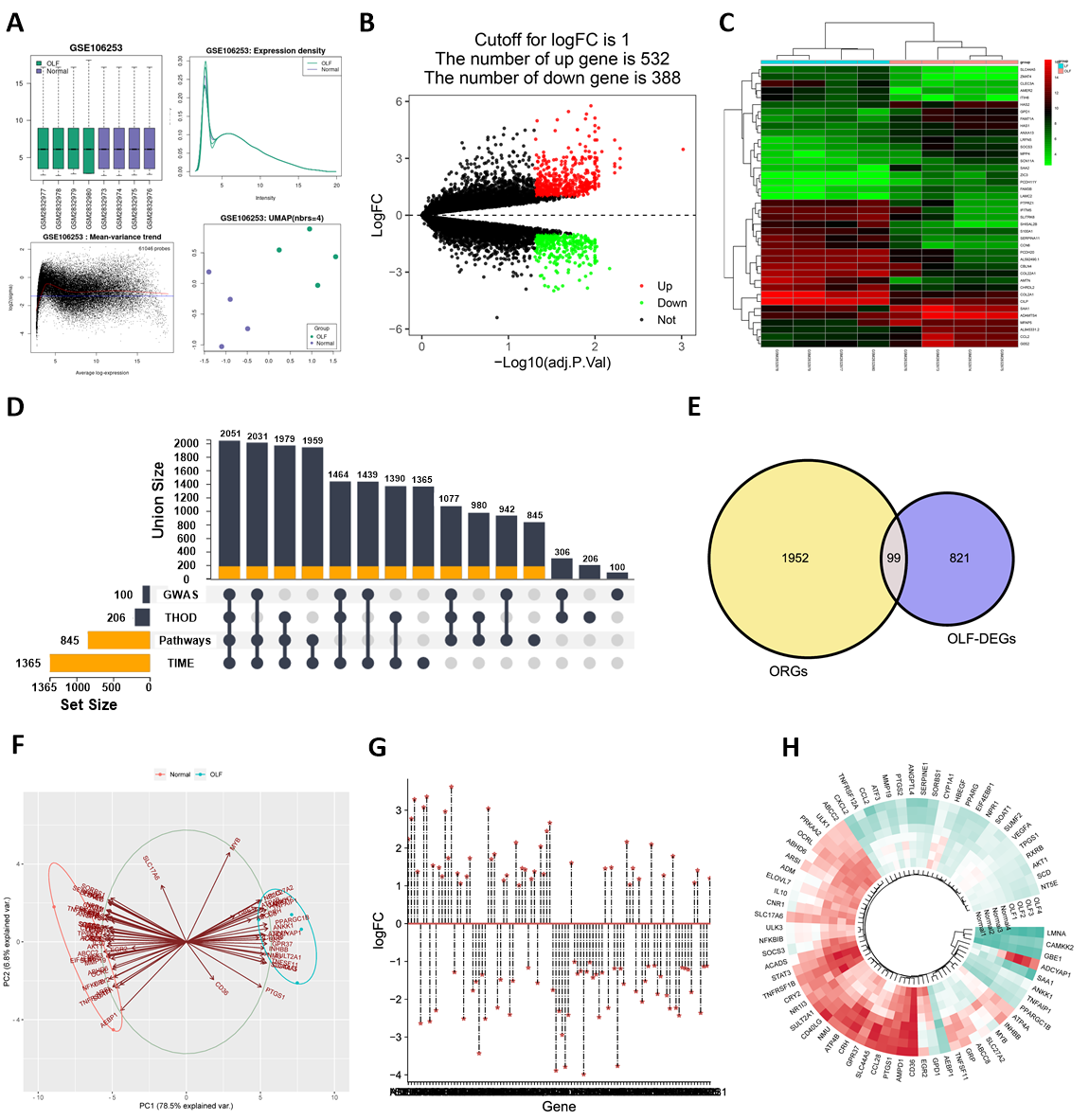


**Supplementary Figure 2.** Key signaling pathways with enriched ORDEGs identified in OLF. (A) The map of the adipocytokine signaling pathway. (B) The map of the TNF signaling pathway. (C) The map of the AMPK signaling pathway. (D) The map of the mTOR signaling pathway. (E) The map of the JAK-STAT signaling pathway.


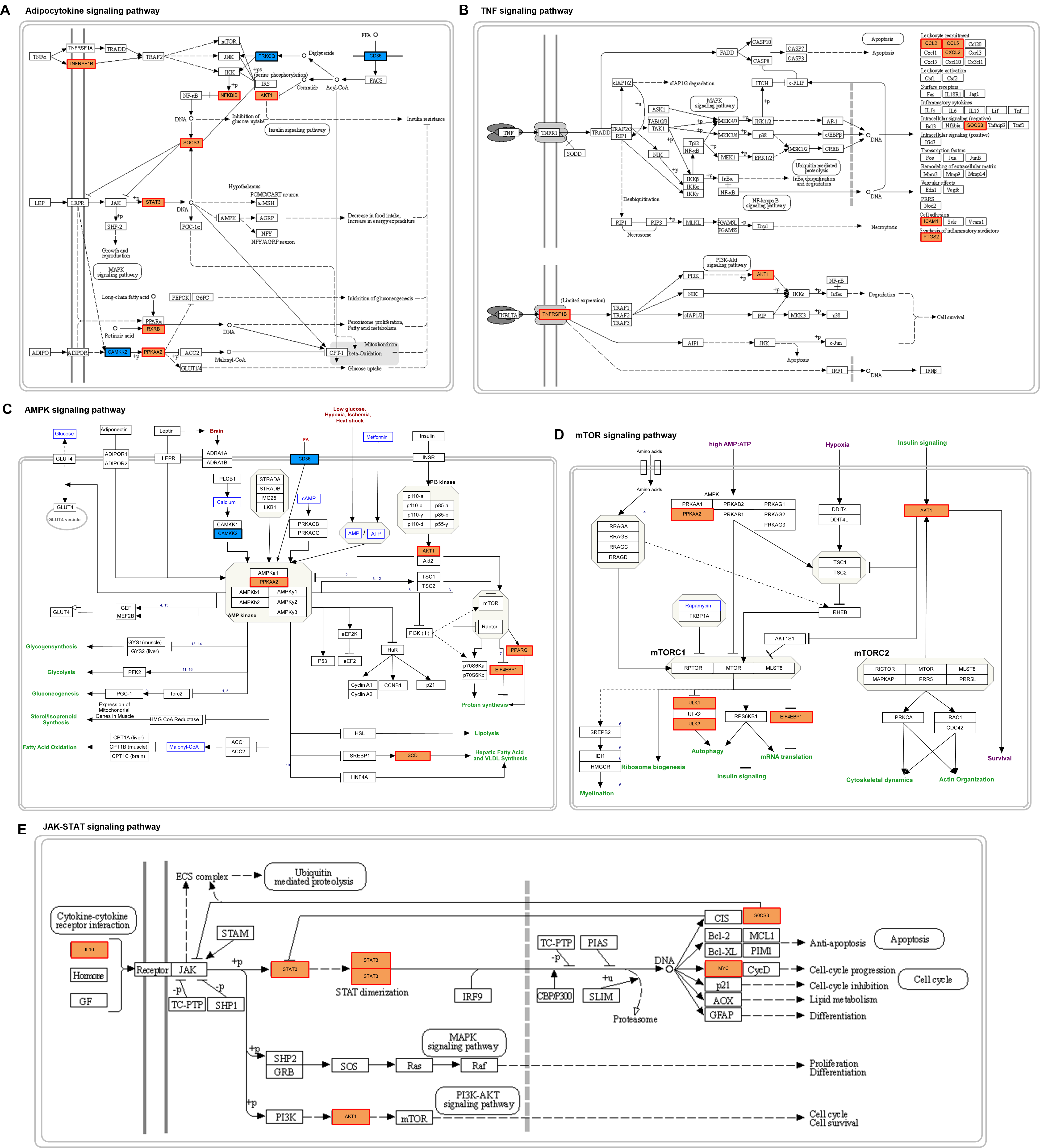


**Supplementary Figure 3.** Topology analysis of the PPI network included clustering coefficient, average shortest path length, betweenness centrality and closeness centrality.


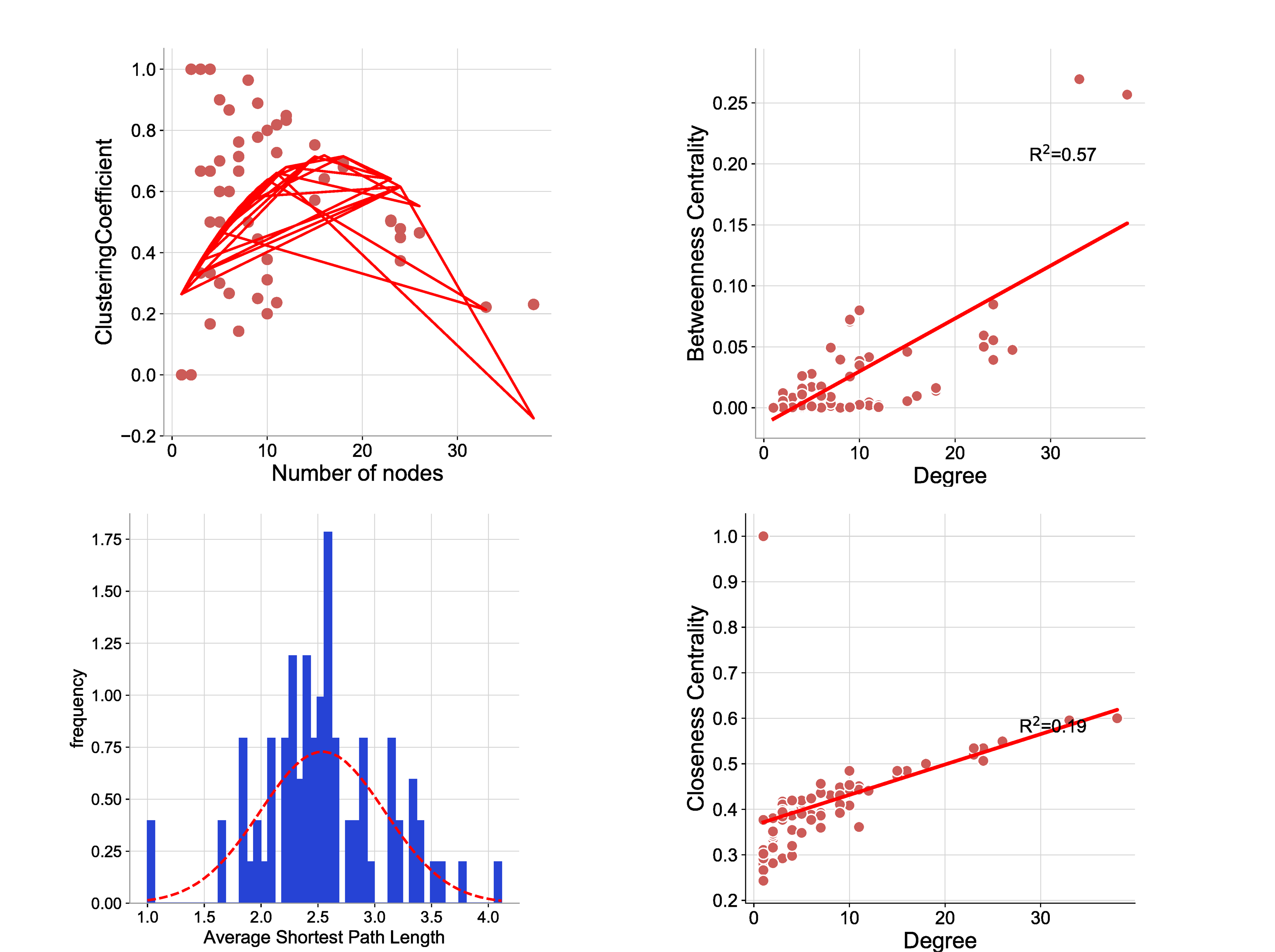


## Supplementary Tables

**Supplementary Table 1.** Basic clinical data of included patients from OLF group and normal group.

| OLF group | | | | | Normal group | | | | |
| --- | --- | --- | --- | --- | --- | --- | --- | --- | --- |
| Patient | Diagnosis (location) | Gender | Age (years) | BMI (kg/m^2^) | Patient | Diagnosis (location) | Gender | Age (years) | BMI  (kg/m^2^) |
| 1 | TOLF (T10-11) | Male | 53 | 23.88 | 1 | TDH (T11-12) | Female | 65 | 26.35 |
| 2 | TOLF (T11-12) | Male | 64 | 26.87 | 2 | TF (T6-9) | Male | 70 | 29.41 |
| 3 | TOLF (T10-12) | Female | 60 | 28.52 | 3 | TDH (T10-12) | Male | 62 | 31.70 |
| 4 | TOLF (T11-12) | Male | 73 | 24.77 | 4 | TF (T12) | Male | 48 | 19.53 |
| 5 | TOLF (T8-12) | Male | 62 | 23.88 | 5 | TDH (T10-12) | Male | 76 | 25.97 |
| 6 | TOLF (T1-3, T6-9) | Male | 63 | 31.02 | 6 | TDH (T9-11) | Male | 53 | 28.41 |
| 7 | TOLF (T1-5) | Female | 53 | 25.25 | 7 | TDH (T11-12) | Female | 61 | 30.44 |
| 8 | TOLF (T10-12) | Male | 64 | 33.67 | 8 | TF  (T10, T12) | Male | 54 | 24.49 |
| 9 | TOLF (T11-12) | Male | 60 | 32.04 | 9 | TDH (T11-L1) | Female | 58 | 29.30 |
| 10 | TOLF (T8-10) | Female | 53 | 27.70 | 10 | TDH (T8-10) | Male | 61 | 25.26 |

**Supplementary Table 2.** The primer sequence used for qRT-PCR in this study.

| Gene Name | Forward 5’-3’ | Reverse 5’-3’ |
| --- | --- | --- |
| SOCS3 | AGAGCGGATTCTACTGGAGCG | CTGGATGCGTAGGTTCTTGGTC |
| PPARG | TGATGTCTTGACTCATGGGTGT | CACGGAGCTGATCCCAAAGT |
| ICAM-1 | GGAAGGGAGCCAAGTAACTGTGAAG | GAGCGGCAGAGCAAAAGAAGC |
| VEGFA | CTGCTCTCTTGGGTGCACTGG | CACCGCCTTGGCTTGTCACAT |
| MYC | GTGCATCGACCCCTCGGTGG | TTGCGAGGCGCAGGACTTGG |
| CCL2 | TGTCCCAAAGAAGCTGTGATC | ATTCTTGGGTTGTGGAGTGAG |
| NT5E | TCTTCTAAACAGCAGCATTCC | CATTTCATCCGTGTGTCTCAG |
| PTGS2 | GGAGCATAACAGAGTGTGTGATGTG | AAGTATTAGCCTGCTCGTCTGGAAT |
| GAPDH | GACAGTCAGCCGCATCTTCT | GCGCCCAATACGACCAAATC |

**Supplementary Table 3.** Detail information of 18 hub ORDEGs.

| Gene symbols | Full names | Gene function | logFC | Regulation |
| --- | --- | --- | --- | --- |
| CCL2 | C-C motif chemokine 2 | Chemotactic factor that attracts monocytes and basophils but not neutrophils or eosinophils. | -3.99 | Down |
| CD40LG | CD40 Ligand | Costimulates T-cell proliferation and cytokine production. Induces the activation of NF-kappa-B. | 2.67 | up |
| SOCS3 | Suppressor of cytokine signaling 3 | SOCS3 is involved in negative regulation of cytokines that signal through the JAK/STAT pathway. | -3.43 | Down |
| CXCL2 | C-X-C motif chemokine 2 | Produced by activated monocytes and neutrophils and expressed at sites of inflammation. | -2.31 | Down |
| IL10 | Interleukin-10 | Inhibits the synthesis of a number of cytokines, including IFN-gamma, IL-2, IL-3, TNF and GM-CSF produced by activated macrophages and by helper T-cells. | -2.29 | Down |
| PTGS2 | Prostaglandin G/H synthase 2 | PTGS2 is responsible for production of inflammatory prostaglandins. | -2.25 | Down |
| STAT3 | Signal transducer and activator of transcription 3 | Signal transducer and transcription activator that mediates cellular responses to interleukins, KITLG/SCF, LEP and other growth factors. Acts as a regulator of inflammatory response. | -1.79 | Down |
| SOCS1 | Suppressor of cytokine signaling 1 | SOCS1 is involved in negative regulation of cytokines that signal through the JAK/STAT3 pathway. | -1.76 | Down |
| TNFRSF1B | Tumor necrosis factor receptor superfamily member 1B | This receptor mediates most of the metabolic effects of TNF-alpha, and regulates TNF-alpha function by antagonizing its biological activity. | -1.32 | Down |
| AKT1 | RAC-alpha serine/threonine-protein kinase | It regulates many processes including metabolism, proliferation, cell survival, growth and angiogenesis. | -1.27 | Down |
| ICAM1 | Intercellular adhesion molecule 1 | CAM proteins are ligands for the leukocyte adhesion protein LFA-1 (integrin alpha-L/beta-2). | -1.21 | Down |
| MYC | Myc proto-oncogene protein | Binds to the VEGFA promoter, promoting VEGFA production and subsequent sprouting angiogenesis. | -1.19 | Down |
| VEGFA | Vascular endothelial growth factor A | Growth factor active in angiogenesis, vasculogenesis and endothelial cell growth. | -1.18 | Down |
| CCL5 | C-C motif chemokine 5 | Chemoattractant for blood monocytes, memory T-helper cells and eosinophils. Causes the release of histamine from basophils and activates eosinophils. | 1.38 | Up |
| NT5E | 5'-Nucleotidase Ecto | Hydrolyzes extracellular nucleotides into membrane permeable nucleosides. Exhibits AMP-, NAD-, and NMN-nucleosidase activities. | -1.18 | Down |
| PPARG | Peroxisome Proliferator Activated Receptor Gamma | It controls the peroxisomal beta-oxidation pathway of fatty acids. Key regulator of adipocyte differentiation and glucose homeostasis. | -1.16 | Down |
| SERPINE1 | Serpin Family E Member 1 | As PLAU inhibitor, it is involved in the regulation of cell adhesion and spreading. | -2.64 | Down |
| TNFSF11 | TNF Superfamily Member 11 | Osteoclast differentiation and activation factor. Augments the ability of dendritic cells to stimulate naive T-cell proliferation. | 3.04 | Up |

**Supplementary Table 4.** Correlation analysis between SOCS3 and immune cell marker gene in OLF.

| Immune cells | Gene markers | Correlation | P |
| --- | --- | --- | --- |
| B cell | CD19 | -0.32 | 0.44 |
|  | CD79A | -0.092 | 0.83 |
|  | MS4A14 | -0.11 | 0.79 |
|  | CD70 | 0.32 | 0.44 |
| T cell | CD3D | -0.75 | 0.032* |
|  | CD2 | -0.73 | 0.041* |
| CD8 + T cell | CD8B | -0.89 | 3.2e-02* |
|  | IL2RA | 0.38 | 0.35 |
| Tfh | CXCR3 | -0.034 | 0.94 |
|  | IL21 | -0.25 | 0.54 |
|  | ICOS | 0.83 | 0.011* |
| Th1 | T-bet (TBX21) | 0.28 | 0.5 |
|  | IL12RB1 | 0.48 | 0.22 |
|  | STAT1 | 0.39 | 0.34 |
|  | TNF-α (TNF) | 0.56 | 0.15 |
| Th2 | CCR4 | 0.68 | 0.062 |
|  | STAT6 | 0.92 | 0.001* |
|  | HAVCR1 | -0.26 | 0.54 |
| Th17 | STAT3 | 0.98 | 9.5E-06* |
|  | IL17A | 0.16 | 0.71 |
|  | IL21R | -0.43 | 0.28 |
|  | IL23R | 0.049 | 0.91 |
| Treg | FOXP3 | 0.68 | 0.065 |
|  | NT5E | 0.89 | 0.0029* |
|  | IL7R | -0.091 | 0.83 |
|  | TGFβ (TGFB1) | -0.74 | 0.034* |
| T cell exhaustion | PD-1 (PDCD1) | 0.13 | 0.77 |
|  | CTLA4 | -0.37 | 0.37 |
|  | LAG3 | -0.84 | 8.3e-03* |
| M1 Macrophage | INOS (NOS2) | 0.59 | 0.12 |
|  | IRF5 | 0.12 | 0.77 |
|  | COX2 (PTGS2) | 0.93 | 9.3e-04* |
| M2 Macrophage | CD163 | 0.27 | 0.52 |
|  | VSIG4 | 0.88 | 4.4e-03* |
|  | MS4A4A | 0.0097 | 0.98 |
|  | MRC1 | 0.57 | 0.14 |
|  | CD209 | 0.043 | 0.92 |
| Monocyte | CD33 | -0.39 | 0.34 |
|  | CD115 (CSF1R) | -0.53 | 0.18 |
| Natural killer cell | CD7 | 0.15 | 0.73 |
|  | KIR2DL4 | -0.22 | 0.61 |
|  | KIR3DL1 | 0.17 | 0.69 |
|  | KIR3DL2 | -0.53 | 0.18 |
|  | KIR2DS4 | 0.42 | 0.3 |
| Neutrophil | CD55 | 0.47 | 0.24 |
|  | CD11b (ITGAM) | 0.54 | 0.17 |
|  | CCR7 | 0.33 | 0.42 |
| Dendritic cell | THBD | 0.58 | 0.13 |
|  | HLA-DPB1 | -0.26 | 0.54 |
|  | HLA-DRA | -0.065 | 0.8 |
|  | HLA-DPA1 | -0.21 | 0.62 |
|  | BDCA-1 (CD1C) | -0.97 | 9.3e-05* |
|  | BDCA-4 (NRP1) | 0.89 | 2.9e-03* |
|  | CD11c (ITGAX) | -0.47 | 0.24 |
